# Patient-reported outcome measures (PROMs) and clinical decision-making in inflammatory rheumatic conditions: a systematic review

**DOI:** 10.1093/rap/rkag093

**Published:** 2026-07-23

**Authors:** Jake Grange, James A Prior, Jon Packham, Max Somer, Helen Parsons, Kirstie L Haywood

**Affiliations:** Warwick Medical School, University of Warwick, Coventry, UK; Department of Rheumatology, Haywood Hospital, Midlands Partnership University NHS Foundation Trust, Staffordshire, UK; School of Medicine, Keele University, Staffordshire, UK; Department of Rheumatology, Haywood Hospital, Midlands Partnership University NHS Foundation Trust, Staffordshire, UK; School of Medicine, Keele University, Staffordshire, UK; Warwick Medical School, University of Warwick, Coventry, UK; Warwick Medical School, University of Warwick, Coventry, UK; Warwick Medical School, University of Warwick, Coventry, UK

**Keywords:** patient-reported outcome measures (PROMs), clinical decision-making, RA, PsA, axSpA, review

## Abstract

**Objectives:**

To examine the role of patient-reported outcome measures (PROMs) in clinical decision-making across three inflammatory arthritides: RA, PsA and axial SpA (axSpA).

**Methods:**

We conducted a systematic review of four online medical literature databases (MEDLINE, Embase, CINAHL, Cochrane Central) from inception to February 2025. Abstracts and full texts were independently double-assessed. Articles examining the use and influence of PROMs on RA, PsA and axSpA clinical decision-making were included, with extracted data narratively synthesised (PROSPERO registration: CRD42024550525).

**Results:**

From 16 950 identified studies, 29 were included (*n* = 22 RA, *n* = 4 axSpA, *n* = 3 PsA; study design: *n* = 15 trials, *n* = 10 cohort, *n* = 4 cross-sectional). PROMs supported three aspects of healthcare: treatment decision-making (*n* = 24), clinical encounters (*n* = 2) and self-monitoring (*n* = 6). Treat-to-target studies (*n* = 14/24, commonly RA) used PROMs to guide drug alterations. However, clinicians adhered less to PROM-based treatment protocols for complex patients or those with restricted insurance policies. PROMs improved patient communication and trust in clinicians. PROM-based self-monitoring matched standard care outcomes, but service limitations reduced its efficiency.

**Conclusion:**

PROMs capture valuable patient data and show potential to support clinical decision-making, particularly in RA patients. Current PROM implementation methods are varied. Standardisation could maximise the clinical value of PROM data that, if acted upon, could enhance patient care and outcomes. However, service and clinician barriers, combined with poor standardisation, currently risk these data being underutilised.

Key messages

- PROMs show promise in supporting three areas of rheumatology care, but barriers mitigate their influence.
- Inadequate standardisation of PROM implementation in rheumatology practice risks clinicians underutilising valuable patient data.
- Service or training limitations can hinder clinicians’ ability to collect and act on PROM data.

## Introduction

RA, axial SpA (axSpA) and PsA are among the most common inflammatory arthritides [1], affecting an estimated 0.46%, 0.12% and 0.67% of the global population, respectively [2–7]. These conditions cause inflammation and pain that requires long-term management [8–10]. Patients also experience individualised health challenges, including stiffness, fatigue, mood disturbances and reduced social functioning [11–13]. Accurate disease assessment is essential to the optimisation of disease management and patient outcomes [14].

Monitoring inflammatory arthritis traditionally involves clinician-reported outcome measures (CROMs), such as inflammatory marker tests or tender/swollen joint counts that infer active disease and guide treatment decisions [15, 16]. However, these provide limited insights into the more subjective health experiences often prioritised by patients, including pain and fatigue, the neglecting of which could risk suboptimal care [17]. Patient-reported outcome measures (PROMs) are increasingly recommended across rheumatological conditions, providing a patient-derived assessment of how they feel, function and respond to treatment [17]. Outcome-reporting guidance, including core domain sets developed by the OMERACT, align with the adoption of specific PROMs [e.g. visual analogue scale (VAS) for pain in RA [18], PsA impact of disease (PSAID) questionnaire [19] and BASDA I [20]]. While widely used in research, PROMs are increasingly used in routine practice settings, supporting the long-term monitoring of patients. However, their influence on clinical decision-making and care outcomes is unknown.

Composite, or hybridised measures, combine several measures into one ‘index’, with the suggestion that they may provide a better illustration of disease than a single measure [14]. For example, the clinical and simplified disease activity indices (CDAI and SDAI, respectively) collect both CRO-specific and PRO-related data [21]. These tools can also be used in dual-target interventions, where PROM and CROM data are used to pursue multiple treatment goals. However, interpretation of such multidimensional assessments can complicate clinical decision-making [22], e.g. where clinical and patient-reported indices improve at different rates or when certain domains improve more than others during treatment [14].

The added value of PRO data in routine practice has been evidenced in clinical oncology, where findings suggest that incorporation can improve patient outcomes [23, 24], care satisfaction [25], patient self-monitoring [26] and shared decision-making [27]. PROMs have also been used to guide individual care and monitor progress in psychiatry [28]. In these contexts, the interventional properties of PROMs are described, e.g. triggering healthcare appointments or aiding patient self-monitoring. However, barriers to their adoption include inadequate technological infrastructure and interpretative guidance [29], which may reduce their value in clinical practice. Equivalent findings regarding the implementational properties of and barriers effecting rheumatology PROMs are lacking.

The growth in virtual interventions such as telemedicine and the movement towards patient-initiated follow-up (PIFU) across rheumatology settings has led to increased use of electronic PROMS (e-PROMs) or hybrid measures [30, 31]. However, the relative value of PROMs in rheumatology practice is unclear. Therefore, this study aimed to review the impact of PROM completion on clinical decision-making and care delivery outcomes for three common rheumatological conditions in routine practice settings.

## Methods

A PICO framework (patient, problem or population; intervention; comparison, control or comparator; outcome) (Supplementary Table S1) [32] underpinned a systematic review of studies that evaluated the impact of PROM completion by patients with RA, axSpA or PsA on clinical decision-making or care delivery outcomes. Composite or hybrid measures—i.e. assessments that combine patient self-reports and clinical tests [e.g. the 28-joint DAS (DAS28)] [33] or report a combination of PROMs with CROMS (e.g. blood tests or imaging)—were also included. Measures without a PROM component were excluded from the analysis. Primary outcomes included any change in clinical decision-making or care delivery that was driven by PROM or hybrid-measure data. Qualitative studies and those allowing patient proxies to complete measures were excluded [34].

The review was registered with the International Prospective Register of Systematic Reviews (PROSPERO; CRD42024550525) and is reported as per the Preferred Reporting Items for Systematic Reviews and Meta Analyses (PRISMA) guidance [35].

### Search strategy

Four online medical databases were searched (EMBASE, Medline, CINAHL, CENTRAL) from inception to 1 February 2025 (Supplementary Table S2). Search terms were developed iteratively from similar reviews in other disciplines [36] to identify studies that described the use of PROMs (or hybrid measures) to inform clinical decision-making in line with the chosen PIFU framework (Supplementary Table S1). The final strategy included different naming conventions for the three arthritides, common and disease-specific rheumatology PROMs and terms capturing broad alterations in care processes. All titles were initially screened by a single author (J.G.). Three authors then independently reviewed abstracts (J.G., J.A.P., M.S.) and full texts (J.G., J.A.P., J.C.P.). Disagreement was resolved through group discussion.

### Data extraction and synthesis

Extracted data included study characteristics (author, publication year, setting, location), study design, patient population, sample size, PROM and intervention detail. Outcomes of interest included care decision-making, health status, patient–clinician relationships, treatment satisfaction and treatment adherence related to PROM use. All data were extracted by J.G. and independently validated by J.A.P.

Synthesis was based on synthesis without meta-analysis (SWiM) guidance informing the study grouping [37], with narrative synthesis used to report the results [38].

### Quality assessment

Two authors (J.G., J.A.P.) independently completed a quality assessment of each included study using an adapted version of the Newcastle–Ottawa Scale (NOS) [39, 40]. Any disagreement was explored with a third author (J.C.P.).

## Results

### Study characteristics

From 16 950 identified studies, 3132 duplicates were removed. After title screening, 742 abstracts were reviewed, with 51 studies eligible for full-text review and forward reference searching. A total of 29 remaining studies were included (Table 1), including randomised controlled (*n* = 12), non-randomised (*n* = 3), cohort (*n* = 10) and cross-sectional (*n* = 4) studies. Studies varied in location, setting and sample (Table 2); most were European (*n* = 25) and recruited people with RA (*n* = 22).

**Table 1:** Reviewed studies: study information (*N* = 29).

Disease	Author	Year	Aim	Study design
RA	Grigor [54]	2004	To test whether an intensive outpatient management program for patients with RA would improve outcomes compared with standard care	RCT
	El Miedany [43]	2012	To assess the integration of PROMS and patient education using joint fitness program and the effect of this combo on disease activity and therapy adherence	RCT
	Dougados [42]	2014	To evaluate the impact of a nurse led programme on comorbidities and the impact of patient self-assessment of disease activity index in RA	RCT
	Akdemir [57]	2016	To compare rheumatologist’s adherence to treatment protocols for RA when using DAS <2.4 or <1.6 as a target	RCT
	de Thurah [69]	2018	To test whether PRO-based telemedicine is comparable to standard care and to compare PRO-led telemedicine delivered by nurses and doctors	RCT
	Desai [49]	2021	To integrate T2T for RA through routine e-PROMs and a multidisciplinary learning intervention for rheumatologists	RCT
	Seppen [67]	2022	To compare app-steered care for low-intensity RA with standard care	RCT
	Gedert [63]	2024	To assess the feasibility of incorporating the PROMIS into rheumatology practice	RCT
	de Jong [41]	2012	To explore whether different PROMs (if considered) would lead to different levels of treatment change	Stratified single-blinded trial
	Fredriksson [68]	2016	To test the hypothesis that implementing a patient-initiated system of care could improve clinical outcomes for RA using disease activity–guided management	Blinded end-point study
	Soubrier [51]	2006	To see which factors best predict a change in DMARD	Cohort study
	Goekoop-Ruiterman [55]	2010	To compare DAS-driven care to standard care	Cohort study
	Schipper [46]	2012	To compare tight control to regular care for early RA	Cohort study
	Farman [52]	2015	To assess the approach of T2T in daily clinical practice for RA	Cohort study
	Brenol [62]	2015	To test the feasibility of T2T in RA	Cohort study
	Steunebrink [53]	2016	To examine predictors of remission in a real-life T2T cohort	Cohort study
	Yun [56]	2020	To identify which measures clinicians actually use in practice and identify how discordant disease activity measurement using different tools impacts RA treatment changes	Cohort study
	Seppen [50]	2022	To assess the probability that patients with low disease activity scores on their e-PROMs will not need a DMARD or steroid intensification	Cohort study
	Solomon [66]	2023	To see whether integrating a PRO-based mobile app for RA patients leads to a reduction in appointments	Cohort study
	van den Dikkenberg [48]	2024	To assess whether e-PROM scores could serve as a triage tool before consultations	Cohort study
	Dougados [42]	2013	To assess the relative weight of PROMs *vs* CROMs on DMARD intensification in RA	Cross-sectional
	Gavigan [44]	2020	To understand barriers to optimal treatment of RA	Cross-sectional
PsA	Coates [58]	2015	To assess T2T in PsA	RCT
	Gazitt [45]	2021	To test the implementation of T2T in PsA	Cohort study
	Coyle [65]	2024	To explore whether PROMs influence care decisions and patient satisfaction	Cross-sectional
axSpA	Molto [59]	2021	To compare T2T to usual care for axSpA	RCT
	López-Medina [60]	2024	To assess protocol deviations in axSpA T2T	RCT
	Beckers [61]	2021	To evaluate the extent to which internationally agreed T2T recommendations were applied in clinical practice in patients with axSpA	Cohort study
	von Rohr [47]	2023	To investigate student-led clinics and e-PROMs to accelerate diagnosis and treatment for axSpA	Cohort study

**Table 2:** Study settings, locations and sample sizes.

Characteristics		RA	PsA	axSpA	Total
Total studies, *N*		22	3	4	29
Setting	District hospitals	19	3	3	25
	Primary care	–	–	1	1
	Not specified	3	–	–	3
Recruitment location	Netherlands	7	–	1	8
	USA	3	–	–	3
	France	2	–	–	2
	United Kingdom	1	1	–	2
	Multiple countries (Europe)	–	1	1	2
	Brazil	1	–	–	1
	Denmark	1	–	–	1
	Germany	–	–	1	1
	Pakistan	1	–	–	1
	Sweden	1	–	–	1
	Not specified	3	1	–	4
	Cross-sectional study	2	1	–	3
Total recruited, *N*	Not given	2	–	–	2
	<100	1	–	1	2
	101–200	5	1	1	7
	201–300	5	1	–	6
	301–500	1	–	1	2
	≥500	6	1	–	7

### Data synthesis

Studies utilised 27 PROMs, 6 hybrid measures and 17 CROMs (Supplementary Table S3). PROMs were used in three aspects of care: treatment decisions (*n* = 24 studies), clinical encounters (*n* = 2 studies) and self-monitoring (*n* = 6 studies) (Table 3). There was substantial heterogeneity across the included studies with regards to PROM implementation (e.g. online *vs* in person), choice of measures (e.g. specific PROMs or CROMs) and outcomes assessed. Primary study outcomes included changes in disease activity (*n* = 11), treatment adherence (*n* = 9), treatment satisfaction (*n* = 5), patient–clinician relationships (*n* = 2), patients health-related quality of life (*n* = 1) and time to diagnosis (*n* = 1) (Table 3).

**Table 3:** Results: PROMS influence across three aspects of care.

Aspects of care	Disease	Author	Measures used	Relevant outcomes	Do findings favour PROM influence?
Impact of PROMs on treatment decisions	RA	Akdemir (2017) [57]	DAS28	DAS28 is more influential to clinicians pursuing low disease activity rather than remission	Positive
	RA	Farman (2015) [52]	DAS28	59% achieved low disease activity after 6 months of T2T	Positive
	RA	Goekoop-Ruiterman (2010) [55]	DAS28	T2T was deemed superior to standard care for remission	Positive
	RA	Schipper (2012) [46]	DAS28, HAQ	55% of tight control patients had DAS28 remission at study’s end compared with 30% in standard care group	Positive
	RA	Steunebrink (2016) [53]	DAS28	77.2% achieved remission at least once	Positive
	RA	Grigor (2004) [54]	DAS, HAQ	Greater DAS reduction for PROM group *vs* control	Positive
	RA	De Jong (2012) [41]	DAS, DAS-CRP, SDAI, CDAI	Composite PROMs increased treatment intensification *vs* DAS	Positive
	RA	Brenol (2015) [62]	DAS18, CDAI, HAQ	T2T improved low disease activity and remission rates	Positive
	RA	Dougados (2013) (a) [42]	RAID, HAQ, PASS	PROMs influenced 61% of decisions *vs* 42% for CROMs. Younger, hospital-based doctors utilised PROMs more during practice	Positive
	RA	Dougados (2013) (b) [64]	DAS28 (self-assessed), RAID, HAQ	More medication changes occurred in the self-assessment group who calculated their own DAS28 to self-monitor	Positive
	RA	van den Dikkenberg (2024) [48]	RAID	Influenced clinician decisions to allocate to telemedicine *vs* standard care	Positive
	RA	Seppen (2022) (a) [50]	RAPID3	100% predictive accuracy for no treatment change over 3 months	Positive
	RA	Gedert (2025) [63]	PROMIS	Increased documentation and PROM-driven changes	Positive
	RA	Gavigan (2020) [44]	PROMIS-CAT, RAPID3	RAPID3 did not match self-assessment; clinician drove decisions	Negative
	RA	Yun (2020) [56]	RAPID3, CDAI, DAS28-ESR, DAS28-CRP	T2T was less effective in moderate–high disease impact	Negative
	RA	Desai (2021) [49]	RAPID3	52% T2T protocol adherence; barriers included comorbidity and perceived progress	Mixed
	RA	Soubrier (2006) [51]	SDAI, DAS	Variables predictive of change were swollen joints, morning stiffness, CRP, tender joint count and global assessment. Composite values associated with the decision to modify DMARDs was DAS28-ESR 4.2, DAS28-CRP 3.6, but SDAI 15 was best	Mixed
	PsA	Coates (2014) [58]	PASI	Higher remission odds (OR 1.91) in T2T group but more adverse events due to medication swapping dictated by measures	Mixed
	PsA	Coyle (2024) [65]	PSAID-12, HAQ	PSAID associated with treatment escalation; limited influence on decisions to reduce medication dosage	Mixed
	PsA	Gazitt (2021) [45]	DAPSA	Clinicians’ decisions aligned with PROM recommendations 65% of the time. In cases where clinician decision-making did not align, 30/40 participants’ disease activity was overestimated due to an ignorance of PROM data	Mixed
	axSpA	Molto (2021) [59]	ASDAS	T2T enabled more patients to experience >30% ASDAS-HI improvement than standard care (47.3% *vs* 36.1%)	Positive
	axSpA	López-Medina (2024) [60]	ASDAS	T2T protocol violations common but did not affect outcomes	Negative
	axSpA	Beckers (2022) [61]	ASDAS-HI, BASFI, HAQ, Medical outcomes survey	T2T protocol followed by only 17% of clinicians (ASDAS) and 15% (BASDAI)	Negative
Impact of PROMs on clinical encounters	RA	El Miedany (2012) [43]	Pain score, patient global assessment, DAS28	PROM group had statistically higher medication adherence, fewer procedures and fewer visits for flares. Both groups saw a significant reduction in disease activity and QOL burden	Positive
	axSpA	von Rohr (2023) [47]	BASDAI	Student-led PROM clinics reduced wait times by >2 months	Positive
Impact of PROMs on self-monitoring	RA	de Thurah (2018) [69]	Flare RA instrument (Danish)	PROM-driven care comparable to standard care	Positive
	RA	Fredriksson (2016) [68]	DAS28	No difference in outcomes; more treatment changes in the self-monitoring group	Positive
	RA	Seppen (2022) [67]	RAPID3	Weekly monitoring with PROMs deemed non-inferior to standard care	Positive
	RA	Solomon (2023) [66]	PROMIS (pain, fatigue, function)	Non-significant reduction in appointments for patients self-monitoring with PROMs; alerts often ignored by clinicians	Negative

### Quality assessment

Most studies (*n* = 18/29) were judged to be of acceptable quality according to scores on the NOS (Table 4). Eight studies were limited in outcome reporting [41–48] and four in sample selection [44, 45, 47, 49].

**Table 4:** Study quality assessment.

Author	Sample selection				Comparability	Outcome		
	1	2	3	4	1	1	2	3
Akdemir [57]	b*	a*	a*	a*	No adjustment	b*	a*	b*
Beckers [61]	b*	NA	a*	NA	b*	b*	a*	a*
Brenol [62]	b*	NA	a*	NA	No adjustment	b*	a*	a*
Coyle [65]	a*	NA	a*	NA	b*	b*	NA	NA
de Jong [41]	b*	NA	a*	NA	No adjustment	b*	NA	d
de Thurah [69]	b*	a*	a*	NA	No adjustment	b*	NA	b*
Desai [49]	a*	a*	a*	b	b*	b*	a*	a*
Dougados (a) [42]	a*	NA	a*	NA	a*b*	d	NA	b*
Dougados (b) [64]	a*	a*	a*	a*	a*	b*	a*	b*
el Miedany [43]	b*	a*	a*	a*	No adjustment	c	a*	b*
Farman [52]	b*	NA	a*	a*	No adjustment	b*	a*	b*
Fredriksson [68]	b*	a*	NA	a*	b*	a*	a*	b*
Gavigan [44]	b*	NA	c	NA	No adjustment	c	NA	NA
Gazitt [45]	b*	NA	a*	NA	No adjustment	b*	NA	c
Goekoop-Ruiterman [55]	b*	b	a*	NA	No adjustment	b*	a*	b*
Schipper [46]	a*	a*/b	a*	a*	b*	b*	a*	d
Seppen (a) [50]	b*	a*	a*	a*	a*b*	b*	a*	b*
Seppen (b) [67]	b*	NA	a*	a*	No adjustment	b*	a*	a*
Solomon [66]	b*	a*	a*	NA	No adjustment	b*	a*	b*
Soubrier [51]	b*	NA	a*	NA	No adjustment	b*	a*	a*
Steunebrink [53]	a*	NA	a*	NA	No adjustment	b*	a*	b*
von Rohr [47]	d	NA	b*	NA	No adjustment	c	NA	NA
Yun [56]	a*	NA	a*	a*	a*b*	b*	a*	b*
Coates [58]	a*	a*	a*	a*	b*	b*	a*	b*
Grigor [54]	b*	a*	a*	NA	No adjustment	b*	a*	a*
Molto [59]	a*	a*	a*	NA	a*b*	b*	a*	b*
Lopez-Medina [60]	a*	a*	a*	NA	a*b*	b*	a*	b*
Gedert [63]	a*	a*	a*	a*	a*b*	a*	a*	b*
van den Dikkenberg [48]	a*	a*	a*	NA*	a*b*	a*	a*	c

The NOS assessment form for cohort studies marks studies with a star (*) for each item on the scale they pass. The scale has three sections. Selection refers to the appropriateness of the cohort and has four components examining the exposed group representativeness, selection of the non-exposed cohort, ascertainment of exposure and proof that the outcome of interest was not present at the start of the study. Comparability refers to the ability to compare between study groups. This was not relevant to all included studies. Outcome refers to the ways in which outcomes were derived. This has three components: assessment of outcome, whether the length of follow-up was sufficient and the adequacy of follow-up.

The following synthesis described the implementation and influence of PROMs across the three aspects of care.

### Impact of PROMs on treatment decisions

Across the studies, PROMs influenced treatment decisions and care outcomes by informing personalised care plans and guiding protocolised and non-protocolised treatment adjustments (*n* = 23). Their influence varied between conditions, with research predominantly concerning RA. Studies also identified clinician factors that altered PROM influence.

#### Informing care plans

Two studies demonstrated that PROMs could distinguish between stable patients and those likely to require imminent care modifications [48, 50]. One study applied the Rheumatoid Arthritis Impact of Disease (RAID) PROM to an outpatient cohort of 802 patients, finding it to have good discriminative value between patients with high disease activity who required continued follow-up and those with low disease activity who could be moved to remote monitoring [48]. The second study examined the medical records of 302 patients over 10 months, comparing e-PROM data against rates of DMARD intensification [50]. The results suggested that low Routine Assessment of Patient Index Data 3 (RAPID3) scores were predictive of patients with RA not requiring a medication change for at least 6 weeks post-assessment. If the patient also displayed an absence of flare symptoms, this duration extended to 6 months. Although not exploring PROM influence as a primary outcome, these results suggest that measures can provide clinicians with the necessary information to individualise patient care.

However, a further study suggests that PROMs may be less influential to a clinician’s decision-making than other data sources [51]. In a single-centre prospective cohort study of rheumatology outpatients (*n* = 204), rheumatologists responded to a battery of PROM and CROM data in determining whether to escalate or maintain current care. When invited to rank the variables that most influenced their decisions, clinicians prioritised swollen joint counts, morning stiffness and CRP levels. Due to the incorporation of these variables, the Simplified Disease Activity Index (SDAI), an RA hybrid measure, was also favoured. The authors suggested swollen joint count more readily predicted the need for treatment change than other variables. Of note, the patient’s perspective was not sought.

#### Influencing protocolised and non-protocolised alterations in care

Most studies (*n* = 14) investigated treat-to-target (T2T) interventions, where PROM and hybrid measure fluctuations drive protocolised treatment adjustments until a predetermined goal, e.g. remission, is reached 45^,^^46^^,^^49^^,^^52–63^. While T2T studies were included for each condition, they were most prominent in RA. Five RA trials that utilised PROMs to guide treatment protocols concluded that T2T was superior to standard care, supporting a greater proportion of patients reaching remission across a shorter time frame [46, 52–55]. However, adherence to T2T interventions was described as a substantial concern in other studies, with factors including patient age, complexity, comorbidity and insurance coverage influencing a clinician’s decision to deviate from protocol [49, 56]. Additionally, evidence from a multicentre randomized controlled trial (RCT) of RA patients (*n* = 1109) suggests that clinicians may be more adherent to T2T protocols where the treatment goal is low disease activity compared with remission [57]. This may be influenced by a clinicians’ perception of the PROM’s accuracy in capturing a patient’s health status and if the treatment goal is achievable [57]. This suggests that the influence of PROMs or hybrid measures in T2T protocols may be overshadowed by certain patient variables or the pursuit of more realistic treatment goals.

In PsA, T2T was similarly associated with superior remission rates [odds ratio (OR) 1.91 (CI 1.03, 3.55)], and additionally, higher instances of adverse events due to the regular medication alterations triggered by measurement fluctuations [58]. However, it was unclear whether alterations were made primarily due to fluctuations in PROMs, CROMs or both. Incorrect PROM data interpretation was described as a further barrier to T2T implementation and protocol adherence [45]. From 40 patients who received a treatment change against T2T protocol, 75% (*n* = 30/40) was attributed to an overestimation of patients’ disease intensity due to a misinterpretation of the Disease Activity in Psoriatic Arthritis (DAPSA) Index hybrid measure.

While one axSpA T2T study described the intervention as comparable to standard care [59], similar adherence issues were described in two trials [60, 61]. In the first, protocol violations occurred regularly, with 51% of clinicians deviating from protocol at least once [60]. In the second, protocol-driven medication changes only occurred in 17% of one cohort measured with the Ankylosing Spondylitis Disease Activity Score (ASDAS) and 15% of another measured with the BASDAI [61]. This study attributed these adherence challenges to resource limitations within the service that reduced clinicians’ ability to respond promptly to PROM data, thus limiting its influence over decision-making.

Beyond T2T, the evidence that PROMs can drive treatment changes in non-protocolised interventions is mixed. An observational cohort study of 1107 patients suggested that 61% of clinician decisions to change treatments were attributed to PROM data [42]. Complimentary findings were reported in a PIFU-focused study, where patients with RA (*n* = 131) experienced more medication changes when consultations were initiated due to DAS28 score fluctuations than when consultations were delivered as part of standard care with PROMs as an auxiliary tool [64]. Alternatively, a cohort study of 296 patients suggested that hybrid measures such as the DAS28, Clinical Disease Activity Index (CDAI) and Simple Disease Activity Index (SDAI) were more likely to influence clinical decision-making than PROMs [41]. The same study reported differences in treatment tapering apportioned to different thresholds for disease activity on the DAS28 and SDAI, with patients completing the DAS28 more likely to undergo more frequent treatment tapering.

Other studies reported that high RAPID3 scores rarely influenced clinician decisions to alter medications in non-T2T cohorts due to the measure not aligning with clinicians’ observations of their patients’ illness [44]. One study explored non-T2T interventions outside of RA [65] and reported that the Psoriatic Arthritis Impact of Disease (PsAID) PROM successfully influenced treatment modifications, but only for medication reduction.

### Impact of PROMs on clinical encounters

Two studies described the use of PROMs in clinical encounters, describing their influence on patient–clinician communication and relationships [43, 47]. In RA, an RCT (*n* = 147) offered their intervention group a joint-fitness educational programme and actively incorporated PROMs into consultations. PROM scores supported discussing care goals, current problems and well-being improvements. At 6 months, this group reported that PROMs enhanced their communication, goal setting and understanding of their own condition, strengthening their trust in the clinician’s ability to act on their needs [43]. In axSpA, e-completion of the BASDAI in a triage clinic setting enhanced medical students’ ability to diagnose axSpA, substantially reducing the time to diagnosis following general practitioner referral [from a median of 92 days (interquartile range 63) to 25 days (interquartile range 25)] [47].

### PROMs influencing self-monitoring

The role of PROMs within self-monitoring was reported across four RA studies, which explored their influence on triggering timely access to care [66–69]. In three of these, regular PROM completion triggered access to triage in response to score fluctuations beyond a certain threshold [67–69]. The triage process supported an exploration of patient need and the arrangement of a face-to-face consultation where appropriate. Evidence suggested that outcomes are comparable to standard care, and this was maintained regardless of whether patients consulted nurses or doctors following triage [70]. A further study removed the triage conversation from the process [66]. Rather, when PROM scores fluctuated beyond a specified threshold, scores were incorporated into patients’ electronic health records, which led to the rheumatologist receiving a notification to advance or delay their next appointment. However, just 17% (*n* = 19/108) of notifications to delay and only 3% (*n* = 1/31) to advance an appointment were actioned. The authors proposed that this was due to an insufficient training period for participating rheumatologists and clinicians having fully booked clinics with no room for appointment alterations, which led to them failing to incorporate PROM-based recommendations. Notably, patients were not involved in the decision-making process to hasten or delay appointments.

## Discussion

Across varied routine rheumatology practice settings, the influence of PROMs on clinical decision-making and care delivery outcomes was evidenced across three aspects of care: informing treatment decisions, clinical encounters and through patient self-monitoring. They were most widely used to positive effect in T2T interventions, with scores successfully influencing medication change according to protocolised amendments. Often used to complement CROs, evidence also supported their positive contribution to clinical consultations and active self-monitoring. However, the wide heterogeneity in PROMs, implementation approaches, outcomes assessed and contexts of use limited the data synthesis.

Evidence suggests that PROMs and hybrid measures can positively influence treatment-related decision-making, particularly in RA T2T studies [46, 52–55]. Although primarily used in rheumatology, T2T has been applied in other conditions, including diabetes mellitus (DM) [71] and IBD [72]. However, here, progress is monitored exclusively with CROMs. The combination of PROM and CROM data to inform T2T decision-making ensures that patient’s feelings and functioning is taken into consideration. Given the wide-ranging and enduring impact of DM and IBD on patients’ lives [73–76], such an approach may be of relevance. Recent studies in oncology suggest that PROMs are used to similar effect to influence and inform clinical decision-making, including treatment alterations and appointment initiation [77, 78]. This contributes towards a reduction in disease burden and enhanced quality of life. However, PROMs are also used within oncology settings to identify treatment side effects that inform medication change, thus reducing treatment burden [25, 79]. Despite the high intervention burden associated with many rheumatology drugs [80], such an approach to the use of PROMs was not identified in this review.

A significant portion of rheumatology patients struggle to reach remission due to their patient global assessment reflecting a poor quality of life, even if their disease activity lessens through intervention. T2T interventions would have limited options to support these patients beyond increasing medication doses, risking harmful side effects for minimal gain [81]. Clinicians may therefore ignore PROM data covering areas of functioning that they feel unable to tackle. Recently, a proposal of ‘dual T2T’ has been proposed that would categorise remission separately based on symptom intensity and disease impact [82]. However, this approach is yet to be tested in large-scale clinical trials to determine whether offering more non-medical support alongside a T2T protocol yields superior outcomes.

Several studies evidenced the potential value of PROMs in helping patients to better communicate their needs and experiences. This was particularly beneficial when PROM scores for patients with fewer complexities were shared between patients and clinicians during the consultation [43]. While only described in two studies, PROMs appeared to be less influential in a clinician’s decision-making when managing complex patients. Rather, factors such as comorbidities, extra-articular manifestations, patient age and insurance coverage were more influential [49, 56]. The reasons for a reduced focus on PROM data were not fully explored. However, a recent systematic review of 42 papers that examined the impact of PROMs on clinical encounters reported clinicians showing similar avoidance of PROM data for palliative or multimorbid patients [83]. In these cases, clinicians felt that discussing PROMs could contribute to patient distress that, due to time limitations, could not be appropriately supported. Additional barriers to PROM implementation included a lack of relevance to patient experience and hence a failure to capture what matters to patients [83, 84]. This could unhelpfully shift the focus of consultations away from patient’s priorities. Where PROMs fail to help patients to communicate the nuances of their experience, their willingness to further engage with PROMs is reduced and the potential benefit to consultations is lost. The face and content validity of PROMs—i.e. how well the PROM captures what matters to patients—is increasingly viewed as the most important ‘measurement property’ [85]. The active involvement of patients in the co-production of PROMs is essential to ensuring high-quality, relevant and acceptable PROMs that have real utility in both research and clinical practice settings [86].

Four studies evidenced the value of PROM-based self-monitoring in enhancing timely access to care [66–69]. While outcomes were largely comparable to standard care, the effectiveness of these approaches was limited by service capacity issues and poor appointment flexibility [66]. To address this, a recent study utilised a PIFU model supported by an e-PROM system that alerted patients and clinicians when a review was required [87]. Stable patients were transferred to PIFU and routine appointments were cancelled to reduce clinic burdens. These patients were then encouraged to contact the department only when they felt the need for clinical input. This system saved ≈1500 consultation hours in the study’s final year, shortening wait times for new patients and enabling quicker responses for patients who required consultation forwarding. Successful implementation was supported by the Non-adoption, Abandonment, Scale-up, Spread, Sustainability framework (NASSS) [88], which informed clinician training, changes to service workflows and e-PROM data integration with electronic health records.

Rheumatology patients respond well to PIFU, gaining empowerment and flexibility in managing their care [89], but some patients report concerns regarding when to seek help [90]. Visual feedback systems, underpinned by evidence of minimal change scores in e-PROMs could alert patients and clinicians to important fluctuations in well-being [91], helping patients to decide whether contact is necessary. PIFU facilitated by e-PROMs represents a promising mechanism through which patients self-monitoring can be improved, while also providing data to clinicians that could support share decision-making.

### Strengths and limitations

A narrative synthesis was conducted in line with best-practice guidance to ensure transparency and replicability of findings [37]. This approach allowed otherwise incomparable studies to be synthesised, generating themes that consolidate the findings of a poorly standardised research topic. The synthesis was supported by an assessment of study quality that was compatible with the varied designs and aims of included research [92]. Included studies were largely found to be of high quality.

The large proportion of western, technologically rich countries present in this review likely contributed to findings that highlighted the importance of technology in supporting PROM implementation. As such, these implications may have less relevance to countries that rely on traditional, paper-based administration of PROMs due to technological limitations.

Notably, this review did not seek to explore whether current PROM-based interventions were superior to standard care, thus leaving some findings without real-world comparison. This would be important for future studies attempting to identify whether the influence of PROM data provides a meaningful benefit to the patient experience over standard care.

The developing role of PROMs in rheumatology routine practice has the potential to benefit numerous parts of the patient journey, including self-monitoring, consultations and treatment-related decision-making. Further developments could include using PROMs to monitor treatment side effects in rheumatology, following the success of this approach in other disciplines such as oncology. For patients to feel their value, they should engage with PROMs that accurately depict their health priorities and have confidence that the information they gather will have relevant use in consultations. Moreover, clinicians should be supported in accessing, interpreting and discussing PROMs so their decision-making can be influenced by data that matters to patients. This may require further training for clinicians to recognise the value of patient-reported data in everyday decision-making processes. Additionally, it is essential to support clinicians and patients with the service and technological infrastructure required to reduce the burden associated with utilising PROMs, such as medical devices, changes to the consultation structure or utilisation of PROM data across the multidisciplinary team [47, 66–69, 93]. Special consideration should be given to complex rheumatology patients and their widely recognised unmet needs [94, 95], which can impact negatively on patient–clinician relationships, care satisfaction and patient self-efficacy [96, 97]. This vulnerable patient group are strong candidates for PROM-based interventions that seek to strengthen the patient’s voice and offer the potential to enrich clinical consultations [17].

However, additional knowledge gaps remain, notably whether certain PROMs influence specific clinical decisions and whether current iterations of PROM-based interventions are superior to standard care. Few studies have gathered stakeholder feedback on the implementation of PROMs, limiting our insights into why these tools may be underutilised [98]. Additionally, as artificial intelligence (AI) continues its integration into care systems [99], there is remit to assess whether AI tools can support PROM dissemination and interpretation.

## Conclusion

Current PROM implementation is poorly standardised in rheumatology, with a range of barriers, including interpretation, dissemination and sceptical attitudes towards their value in practice. However, findings suggest a strong basis for the influence of PROMs across the care journey when these barriers are overcome. It is important to continue refining our understanding of how PROM data can support clinical decision-making so that treatments can be personalised to individual’s needs, consultations can be mutually beneficial to patients and clinicians and patients can feel more empowered to monitor their own conditions.

## Supplementary Material

rkag093_Supplementary_Data

## Data Availability

No new data were created via this study. The data underlying this article are available in the article and in its online supplementary materials.
