# Supplementary material for: Patient-reported outcome measures (PROMs) and clinical decision-making in inflammatory rheumatic conditions: a systematic review
Source: Rheumatol Adv Pract. 2026 Jul 23;10(3):rkag093. doi: 10.1093/rap/rkag093 (PMC13484879; doi:10.1093/rap/rkag093)
Supplement: Supplementary file 1 [file rkag093_supplementary_data.docx]

Supplementary Table S1. Review design framework and eligibility criteria

| **Category** | **Inclusion Criteria** | **Exclusion Criteria** |
| --- | --- | --- |
| P: Population | Adults (≥18) with a diagnosis of Axial Spondyloarthritis, Rheumatoid Arthritis, or Psoriatic Arthritis |  |
| I: Intervention | Any care plan that involves longitudinal (n > 2 instances) PROM completion by patients  Studies using a combination of CROMs and PROMs, or hybrid measures containing clinician and patient reported data. |  |
| C: Control Groups | Studies without a control group were eligible. Where a control group was present, it should consist of a group of patients undergoing standard care. |  |
| O: Outcomes | Any change in clinical decision making or care delivery that was in response to a change in PROM score. This could include but was not limited to:  triggering consultations, changing intervention strategies, altering medication doses, or contributing to diagnostic decisions | Studies using proxies to complete PROMs |
| Setting | Community, primary, secondary, or tertiary care. National or international studies. |  |
| Study Designs | Quantitative studies:   - Cohort studies - Case-control studies - Cross-sectional studies - Randomised control trials | Systematic reviews Case studies Case series Narrative reviews/editorials Qualitative studies |
| **Language** | Available in English | Non-English articles which we were unable to translate |
| **Publication Type** | Full-text studies from peer-reviewed journals | Conference abstracts, Dissertations, protocol papers |

Supplementary Table S2: search strategy

| Ovid MEDLINE(R) ALL <1946 to February 1^st^ 2025 | |
| --- | --- |
|  |  |
| 1 | spondylarthritis/ |
| 2 | spondylarthropathies/ |
| 3 | Spondylitis/ |
| 4 | exp axial spondyloarthritis/ |
| 5 | ankylosi*.ti,ab,kf. [pick up only ankylosing or ankylosis, not ankylosed] |
| 6 | spondyl?arthr*.ti,ab,kf. |
| 7 | spondylitis.ti,ab,kf. |
| 8 | AxSpA.ti,ab,kf. |
| 9 | nrAxSpA.ti,ab,kf. |
| 10 | nAxSpA.ti,ab,kf. |
| 11 | (axial adj3 SpA).ti,ab,kf. |
| 12 | arthritis, psoriatic/ |
| 13 | (psoriat* adj5 (arthr* or polyarthr* or poly arthr* or oligoarthr* or oligo arthri* or monoarthr* or mono arthr* or rheumat*)).ti,ab,kf. |
| 14 | PsA.ti,ab,kf. |
| 15 | Arthritis, Rheumatoid/ |
| 16 | RA.ti,ab,kf. |
| 17 | (rheumat* adj3 (arthrit* or diseas* or condition* or nodule*)).ti,ab,kf. |
| 18 | (Inflamm* adj3 (arthrit* or rheumat*)).ti,ab,kf. |
| 19 | or/1-18 |
| 20 | patient reported outcome measures/ |
| 21 | (Patient reported adj (outcome* or assessment* or symptom*)).ti,ab,kf. |
| 22 | (("quality of life" or wellbeing or QoL or HRQoL or HRQL) adj5 (tool* or question* or scale* or instrument* or index or indices or measure* or profile* or assess*)).ti,ab,kf. |
| 23 | (ePROM* or PROM or PROMS).ti,ab,kf. |
| 24 | minim* disease activity.ti,ab,kf. |
| 25 | health status indicators.ti,ab,kf. |
| 26 | ((patient* or self) adj3 (report* or apprais* or rate* or rating* or response* or evaluat* or administer* or assessment)).ti,ab,kf. |
| 27 | (CIAQ or "combined inflammatory arthritis questionnaire").ti,ab,kf. |
| 28 | (BASDAI or "Bath Ankylosing Spondylitis Disease Activity Index").ti,ab,kf. |
| 29 | (BASFAI or "Bath Ankylosing Spondylitis Functional Index").ti,ab,kf. |
| 30 | (AS-DAS or ASDAS or "Ankylosing Spondylitis Disease Activity Score").ti,ab,kf. |
| 31 | (AS-AIMS or "ankylosing spondylitis-arthritis impact measurement scale").ti,ab,kf. |
| 32 | (CASQ or "Combined Ankylosing Spondylitis Questionnaire").ti,ab,kf. |
| 33 | (AsQoL or EASIQoL or "Ankylosing Spondylitis Quality of Life").ti,ab,kf. |
| 34 | (HAQ-S or "Health Assessment Questionnaire-Spondyloarthropathy").ti,ab,kf. |
| 35 | (WASTEd or "Warwick Axial Spondyloarthritis Fatigue and Energy Questionnaire").ti,ab,kf. |
| 36 | (DAS-28 or "disease activity score").ti,ab,kf. |
| 37 | (RAID or "Rheumatoid Arthritis Impact of Disease").ti,ab,kf. |
| 38 | (mRAI or "modified rheumatology attitude index").ti,ab,kf. |
| 39 | (RADAI or "Rheumatoid Arthritis Disease Activity Index" or RADAI-SF or "Rheumatoid Arthritis Disease Activity Index-Short Form").ti,ab,kf. |
| 40 | (PAS-II or "Patient-based Disease Activity Score").ti,ab,kf. |
| 41 | (PR-TJC or "patient reported tender-joint-count").ti,ab,kf. |
| 42 | (PRO-CLARA or "Patient Reported Outcome Clinical Arthritis Activity").ti,ab,kf. |
| 43 | (SPARRA or "symptoms in persons at risk of rheumatoid arthritis").ti,ab,kf. |
| 44 | diabilities of the arm, shoulder and hand outcome measure.ti,ab,kf. |
| 45 | (PASI or "Psoriasis Activity and Severity index" or "Psoriasis Area Severity Index").ti,ab,kf. |
| 46 | (OAPT or "optimal psoriasis assessment tool").ti,ab,kf. |
| 47 | (PSI or "Psoriasis Symptom Inventory").ti,ab,kf. |
| 48 | (PAIP or "psoriatic arthritis impact profile").ti,ab,kf. |
| 49 | (PASE or "psoriatic arthritis screening and evaluation questionnaire").ti,ab,kf. |
| 50 | (P-SIM or "Psoriasis symptoms and impact measure").ti,ab,kf. |
| 51 | (SPI or "simplified psoriasis index").ti,ab,kf. |
| 52 | (IPBOD or "Inverse Psoriasis Burden of Disease Questionnaire").ti,ab,kf. |
| 53 | psoriasis epidemiology screening tool.ti,ab,kf. |
| 54 | (DAPSA or "Disease Activity in Psoriatic Arthritis").ti,ab,kf. |
| 55 | (CPDAL or "preliminary composite disease activity index").ti,ab,kf. |
| 56 | (PsAID or "Psoriatic Arthritis Imapct of Disease questionnaire").ti,ab,kf. |
| 57 | (PASDAS or "Psoriatic Arthritis disease activity score").ti,ab,kf. |
| 58 | Arthritis Impact Measurement Scale.ti,ab,kf. |
| 59 | patient generated index.ti,ab,kf. |
| 60 | or/20-59 |
| 61 | exp Clinical Decision-Making/ |
| 62 | (process* adj2 care).ti,ab,kf. |
| 63 | exp "patient acceptance of health care"/ |
| 64 | Decision Making, Shared/ |
| 65 | shared decision making.ti,ab,kf. |
| 66 | (Clinic* adj2 (change or judgement or "decision making" or plan* or manage* or coordinati* or alter* or adapt* or tailor* or revis* or reevaluat* or evaluat*)).ti,ab,kf. |
| 67 | (Healthcare adj2 (change or judgement or "decision making" or plan* or manage* or coordinati* or alter* or adapt* or tailor* or revis* or reevaluat* or evaluat*)).ti,ab,kf. |
| 68 | (Intervention adj2 (change or judgement or "decision making" or plan* or manage* or coordinati* or alter* or adapt* or tailor* or revis* or reevaluat* or evaluat*)).ti,ab,kf. |
| 69 | (treatment adj2 (change or judgement or "decision making" or plan* or manage* or coordinati* or alter* or adapt* or tailor* or revis* or reevaluat* or evaluat*)).ti,ab,kf. |
| 70 | exp treatment outcome/ |
| 71 | treatment outcome.ti,ab,kf. |
| 72 | or/61-71 |
| 73 | ((randomi*ed controlled trial or controlled clinical trial).pt. or randomi*ed.ab. or placebo.ab. or drug therapy.fs. or randomly.ab. or trial.ab. or groups.ab.) not (exp animals/ not humans.sh.) |
| 74 | randomized controlled trials/ |
| 75 | exp Cohort Studies/ |
| 76 | cohort.ti,ab,kf. |
| 77 | exp case-control studies/ |
| 78 | case control.ti,ab,kf. |
| 79 | Longitudinal Studies/ |
| 80 | longitudinal stud*.ti,ab,kf. |
| 81 | (follow up adj (study or studies)).ti,ab,kf. |
| 82 | prospective.ti,ab,kf. |
| 83 | (observ* adj3 (study or studies)).ti,ab,kf. |
| 84 | health services research/ |
| 85 | or/73-84 |
| 86 | 19 and 60 and 72 and 85 |

Supplementary Table S3. Extracted PROMs, Hybrid Measures, and PROMs

|  | PROM | abbreviation | *RA* | PsA | axSpA | Total |
| --- | --- | --- | --- | --- | --- | --- |
| **PROMs** | Ankylosing Spondylitis Disease Activity Scale | ASDAS |  |  | 2 | 2 |
|  | Bath Ankylosing Spondylitis Disease Index | BASDAI |  |  | 1 | 1 |
|  | Bath Ankylosing Spondylitis Functional Index | BASFI |  |  | 1 | 1 |
|  | Health Assessment Questionnaire | HAQ | 7 |  | 1 | 8 |
|  | Euro-QoL 5-Dimension questionnaire | Euro-QoL EQ-5D |  |  | 1 | 1 |
|  | Medical outcomes survey |  |  |  | 1 | 1 |
|  | Self-efficacy questionnaire |  | 1 |  |  | 1 |
|  | Danish version of the flare-RA instrument |  | 1 |  |  | 1 |
|  | Routine assessment of patient index data 3 | RAPID-3 | 4 | 1 |  | 5 |
|  | Rheumatoid arthritis impact of disease | RAID | 3 |  |  | 3 |
|  | Visual analogue scales | VAS | 7 | 1 |  | 8 |
|  | Patient acceptable symptom state | PASS | 1 |  |  | 1 |
|  | Disease Acticity Scale – 28 self assessment | DAS-28 | 1 |  |  | 1 |
|  | Patient global assessment |  | 2 |  |  | 2 |
|  | Functional disease intensity measure |  | 1 |  |  | 1 |
|  | General Health Related Quality of Life measures |  | 2 |  |  | 2 |
|  | PROMIS-variants |  | 4 | 1 |  | 5 |
|  | Minimal disease activity compostie measures |  |  | 1 |  | 1 |
|  | Disease activity index for psoriatic arthritis | DAPSA |  | 1 |  | 1 |
|  | Perceived efficacy in patient physician interactions - 5 | PEPPI-5 | 1 |  |  | 1 |
|  | Treatment satisfaction questionnaire |  | 2 |  |  | 2 |
|  | General likert scales |  | 1 |  |  | 1 |
|  | Compliance questionnaire for rheumatology | CQR-5 | 1 |  |  | 1 |
|  | Flare questionnaire |  | 1 |  |  | 1 |
|  | RA disease activity index |  | 1 |  |  | 1 |
|  | Patient global disease activity |  |  | 1 |  | 1 |
|  | Short form survey - 36 | SF-36 | 1 |  |  | 1 |
| **hybrid measures** | Disease activity scale – 28 item | DAS-28 | 9 |  |  | 9 |
|  | Disease activity scale – C-reactive protein test | DAS-CRP | 2 |  |  | 2 |
|  | Disease activity scale – 3var | DAS (3var) | 1 |  |  | 1 |
|  | Disease activity scale - Erythrocyte Sedimentation Rate | DAS-ESR | 2 |  |  | 2 |
|  | Simplified disease activity index | SDAI | 2 |  |  | 2 |
|  | Clinical disease activity index | CDAI | 2 |  |  | 2 |
| **CROMs** | Tender joint count |  | 6 | 2 |  | 8 |
|  | Swollen joint count |  | 6 | 2 |  | 8 |
|  | Adverse events counter |  | 1 |  |  | 1 |
|  | General clinical assessment |  | 1 | 1 |  | 2 |
|  | Dactylitis count |  |  | 1 |  | 1 |
|  | Body surface area of psoriasis |  |  | 1 |  | 1 |
|  | Physician rated overall assessment of disease activity score |  |  | 1 |  | 1 |
|  | Widespread pain index and severity scale for fibromyalgia |  |  | 1 |  | 1 |
|  | *CRP blood test* |  | 3 |  | 2 | 5 |
|  | *ESR blood test* |  | 5 |  |  | 5 |
|  | Rheumatoid factor |  | 1 |  |  | 1 |
|  | Anti-cyclic citrullinated peptide |  | 1 |  |  | 1 |
|  | Magnetic resonance imagery | MRI |  |  | 1 | 1 |
|  | X-ray |  |  |  | 1 | 1 |
|  | HLA-B27 antigen test |  |  |  | 1 | 1 |
|  | Tender entheseal points |  |  | 1 |  | 1 |
|  | Psoriasis area and severity index | PASI |  | 1 |  | 1 |

Supplementary Table S4. PRISMA checklist

| **Section/topic** | **#** | **Checklist item** | **Reported on page #** |
| --- | --- | --- | --- |
| **TITLE** | | |  |
| Title | 1 | Identify the report as a systematic review, meta-analysis, or both. | 0 |
| **ABSTRACT** | | |  |
| Structured summary | 2 | Provide a structured summary including, as applicable: background; objectives; data sources; study eligibility criteria, participants, and interventions; study appraisal and synthesis methods; results; limitations; conclusions and implications of key findings; systematic review registration number. | 1 |
| **INTRODUCTION** | | |  |
| Rationale | 3 | Describe the rationale for the review in the context of what is already known. | 4 |
| Objectives | 4 | Provide an explicit statement of questions being addressed with reference to participants, interventions, comparisons, outcomes, and study design (PICOS). | 5 |
| **METHODS** | | |  |
| Protocol and registration | 5 | Indicate if a review protocol exists, if and where it can be accessed (e.g., Web address), and, if available, provide registration information including registration number. | 5 |
| Eligibility criteria | 6 | Specify study characteristics (e.g., PICOS, length of follow-up) and report characteristics (e.g., years considered, language, publication status) used as criteria for eligibility, giving rationale. | 6 |
| Information sources | 7 | Describe all information sources (e.g., databases with dates of coverage, contact with study authors to identify additional studies) in the search and date last searched. | 5 |
| Search | 8 | Present full electronic search strategy for at least one database, including any limits used, such that it could be repeated. | 6, 29 |
| Study selection | 9 | State the process for selecting studies (i.e., screening, eligibility, included in systematic review, and, if applicable, included in the meta-analysis). | 6-7 |
| Data collection process | 10 | Describe method of data extraction from reports (e.g., piloted forms, independently, in duplicate) and any processes for obtaining and confirming data from investigators. | 6-7 |
| Data items | 11 | List and define all variables for which data were sought (e.g., PICOS, funding sources) and any assumptions and simplifications made. | 7, 24 |
| Risk of bias in individual studies | 12 | Describe methods used for assessing risk of bias of individual studies (including specification of whether this was done at the study or outcome level), and how this information is to be used in any data synthesis. | 7 |
| Summary measures | 13 | State the principal summary measures (e.g., risk ratio, difference in means). | NA |
| Synthesis of results | 14 | Describe the methods of handling data and combining results of studies, if done, including measures of consistency (e.g., I^2^) for each meta-analysis. | 7 |

| **Section/topic** | **#** | **Checklist item** | **Reported on page #** |
| --- | --- | --- | --- |
| Risk of bias across studies | 15 | Specify any assessment of risk of bias that may affect the cumulative evidence (e.g., publication bias, selective reporting within studies). | NA |
| Additional analyses | 16 | Describe methods of additional analyses (e.g., sensitivity or subgroup analyses, meta-regression), if done, indicating which were pre-specified. | NA |
| **RESULTS** | | |  |
| Study selection | 17 | Give numbers of studies screened, assessed for eligibility, and included in the review, with reasons for exclusions at each stage, ideally with a flow diagram. | 8, 27 |
| Study characteristics | 18 | For each study, present characteristics for which data were extracted (e.g., study size, PICOS, follow-up period) and provide the citations. | 42 |
| Risk of bias within studies | 19 | Present data on risk of bias of each study and, if available, any outcome level assessment (see item 12). | 13, 28 |
| Results of individual studies | 20 | For all outcomes considered (benefits or harms), present, for each study: (a) simple summary data for each intervention group (b) effect estimates and confidence intervals, ideally with a forest plot. | 42 |
| Synthesis of results | 21 | Present results of each meta-analysis done, including confidence intervals and measures of consistency. | 8-13 |
| Risk of bias across studies | 22 | Present results of any assessment of risk of bias across studies (see Item 15). | 28 |
| Additional analysis | 23 | Give results of additional analyses, if done (e.g., sensitivity or subgroup analyses, meta-regression [see Item 16]). | NA |
| **DISCUSSION** | | |  |
| Summary of evidence | 24 | Summarize the main findings including the strength of evidence for each main outcome; consider their relevance to key groups (e.g., healthcare providers, users, and policy makers). | 13 |
| Limitations | 25 | Discuss limitations at study and outcome level (e.g., risk of bias), and at review-level (e.g., incomplete retrieval of identified research, reporting bias). | 13, 14 |
| Conclusions | 26 | Provide a general interpretation of the results in the context of other evidence, and implications for future research. | 15, 16 |
| **FUNDING** | | |  |
| Funding | 27 | Describe sources of funding for the systematic review and other support (e.g., supply of data); role of funders for the systematic review. | 17 |
